# Prevalence of sexually transmitted infections among cervical cancer suspected women at University of Gondar Comprehensive Specialized Hospital, North-west Ethiopia

**DOI:** 10.1186/s12879-021-06074-y

**Published:** 2021-04-23

**Authors:** Milkias Abebe, Setegn Eshetie, Belay Tessema

**Affiliations:** 1 Department of Medical Laboratory Sciences, Institute of Health Sciences, Wollega University, P.O. Box: 395, Nekemte, Ethiopia; 2 Department of Medical Microbiology, College of Medicine and Health Sciences, School of Biomedical and Laboratory Sciences, University of Gondar, P.O. Box: 196, Gondar, Ethiopia

**Keywords:** HIV, HBV, HCV, Syphilis, Cervical cancer, Ethiopia

## Abstract

**Background:**

Sexually transmitted infections (STIs) such as Human Immunodeficiency Virus (HIV), Hepatitis B Virus (HBV), Hepatitis C Virus (HCV), and Syphilis have continued a significant public health problem, especially in areas with substandard infection prevention and control programs. It is known that STIs are largely associated with the increased occurrence of cervical cancer. To date, little is known about the burden of STIs among cervical cancer suspected women in Ethiopia.

**Objectives:**

To assess the seroprevalence of STIs and associated risk factors among cervical cancer suspected women with special emphasis on HIV, HBV, HCV, and Syphilis.

**Methods:**

A hospital-based cross-sectional study was conducted among cervical cancer suspected women at the University of Gondar Comprehensive Specialized Hospital from February to April 2017. A pre-tested and structured questionnaire was used to collect data on patients’ characteristics. The patient’s serum or plasma samples were tested for HIV, HBV, HCV, and syphilis using enzyme-linked immunosorbent assay. In all aspects, the standard operational procedure was strictly followed. Data were analyzed using SPSS version 20 software and presented using tables. Statistical associations were measured using bivariate and multivariable logistic regression. A *p*-value of below 0.05 was considered statistically significant.

**Result:**

A total of 403 cervical cancer suspected women with the mean age of 42.54 (SD + 11. 24) years were enrolled in the study. The overall prevalence of STIs was 16.6% (67/403) and the prevalence of HIV, HBV, HCV, and syphilis was 36/403 (8.9%), 10/403 (2.5%), 4/403 (1%), and 29/403 (7.2%) respectively. History of multiple sexual partners (Adjusted OR = 3.02, 95%CI 1.57–5.79, *P* = 0.001), alcohol addiction (Adjusted OR = 2.2, 95%CI 1.07–4.5, *P* = 0.031), history of STI (Adjusted OR = 3.38; 95% CI: 1.76–6.47, *P* = 0.00) and not use of condom (Adjusted OR = 4.99; 95% CI: 1.5–16.16, *P* = 0.007) were significantly associated with STIs.

**Conclusion:**

The prevalence of STIs was generally higher among cervical cancer suspected patients. Health education is encouraged to promote awareness about the prevention of STIs.

## Introduction

Sexually transmitted infections are infections caused by pathogens that are mainly transmitted through unsafe sexual activity. They can also spread by non-sexual contact with contaminated blood, tissues, through breastfeeding, and during childbirth. Different types of bacteria, viruses, and parasites can able to cause STIs [1]. Viral STIs like human immunodeficiency virus, human papillomavirus (HPV), hepatitis B virus, and herpes simplex virus are preventable, but not treatable [2]. At present, such STIs (HIV, HBV, and HCV) has continued to be a community health problem in high, middle and low countries that causes acute illness, infertility, long-term disability, and death, with severe medical, and psychological consequences for millions of women and infants [1]. Diferrent reports showed that the prevalence of STIs related with early age of initiation of sexual activity, drug use before sex, and multiple sexual partners [3].

Thought, HPV is a necessary principal cause of cervical cancer, but it is not a sufficient cause [4]. Tobacco smoking, high parity, long-term contraceptive use, and co-infection with HIV and other STIs (syphilis, HBV, and HCV), have been identified as established cofactors necessary for progression from cervical HPV infection to cancer [5]. Women infected with HIV have been shown to have a 2 to 12 fold risk of developing cervical cancer [6]**.** This relationship between HIV infection and HPV infection is also due to the fact that both are sexually transmitted diseases and behaviors that put women at risk for one also put them at risk for the other [6]. Co-infection with other sexually transmitted diseases (syphilis, hepatitis B virus, and hepatitis C virus) and vaginal infections are associated with increased susceptibility to HPV infection [7].

Sexually transmitted diseases, especially HIV, HBV, HCV, and Syphilis infections are the main contributing factors for cervical cancer, which is the second leading cause of cancer death in women [5–7]. Hence, the result of the study is helpful to design a strategy for the prevention of sexually transmitted infection in cervical cancer suspected women, draw the attention of stakeholders to focus on such life-threatening but preventable infections, it also serves as a baseline data for the researchers who are interested in related topics. Therefore, this study aimed to determine the prevalence of sexually transmitted infection and its associated risk factors among women suspected of cervical cancer attending at University of Gondar Comprehensive Specialized Hospital, North-west Ethiopia.

## Methods

### Study design, area, and population

A hospital-based cross-sectional study was conducted among women suspected of cervical cancer from February to April 2017 at the University of Gondar Comprehensive Specialized Hospital. University of Gondar Comprehensive Specialized Hospital is one of the largest comprehensive, specialized hospitals served as a teaching as well as patient care in the Amhara region. It is located in Gondar town, 750 km far from Addis Ababa in Northwest Ethiopia. The hospital has an accredited laboratory, more than 1200 beds, and provides health care referral services for more than 5 million people from the surrounding zones and nearby regions [8].

### Inclusion and exclusion criteria

Cervical cancer suspected women (women screened for cervical cancer who had abnormal vaginal bleeding such as inter-menstrual bleeding, post-coital bleeding) who are attending at University of Gondar Comprehensive Specialized Hospital during the study period were included in the study while patients with known STIs were excluded from the present study.

### Sample size and sampling procedure

The sample size was calculated based on the assumption of 5% expected margins of error and a 95% confidence interval, taking the prevalence of 50% by using a single population proportion formula as follows.

$$ \mathrm{N}=\frac{{\left(\mathrm{Z}\upalpha /2\right)}^2\mathrm{P}\left(1-\mathrm{P}\right)}{d^2} $$

Where N is the calculated sample size of cervical cancer suspected women; Z is the standard normal deviate at 95%, confidence interval = 1.96; P is the prevalence of STI to get maximum sample size = 50%; d is the precision level = 0.05. Then 5% contingency = 19 were added. Finally, the total sample size =384 + 19 = 403 study participants.

The study participants were enrolled consecutively using a convenience sampling technique until a sample size of 403 study participants was achieved. The detailed information of socio-demographic characteristics, behavioral characteristics, clinical and environmental characteristics were collected prior to laboratory diagnosis from each of the study participants using structured and pre-tested questionnaires.

### Laboratory diagnosis

Study participants sera was tested for the presence of sexually transmitted infection (HBV, HIV, HCV and syphilis) by using the Enzyme-Linked Immunosorbent Assay (ELISA). For HIV1/2: Vironostika ELISA (Bio-Merieux, Boxtel, Netherlands); Furthermore, for HBsAg: using ELISA, Hepanostika HBsAg (Bio-Merieux, Boxtel, Netherlands); Moreover, for HCV: Human anti-HCV ELISA (Human Gesellschaft for Bio-chemical and diagnostic MbH, Germany). Besides, anti-syphilis Ab of syphilis was checked by using DIALAB ELISA (Nora Kampitsch, MSc, India). All positive participant results were sent to University of Gondar Comprehensive Specialized Hospital for the appropriate treatment.

### Data analysis and interpretation

Data was collected, summarized, tabulated, and analyzed using Statistical Package Epi-Info Version7 and Statical package for social science version 20 Software. The results were presented through graphs and tables. The statistical significance, the association was measured by using bivariate and multivariate analysis, odds ratio at 95% confidence intervals. A *p*-value < 0.05 was considered as statistically significant.

### Operational definition of term

Positive STIs defined as a positive status either for single or combination of infections specific to HIV, HBV, HCV, and syphilis.

## Results

### Socio-demographic data

A total of 403 cervical cancer suspected women were included in the study. The majority of the respondents were in the age groups of 36–49 years, 179 (44.4%), and the mean age of study subjects was 42.54 + 11.24 years. Nearly 70% of the participants were married, and most of the 230 (57.1%) the participants were unable to read and write and 199 (49.4%) of were housewives, 252 (67.5%) of participants were from the urban (Table 1).

**Table 1:** Socio-demographic characterstics of cervical cancer suspected women at University of Gondar Comprehensive Specialized Hospital

Characteristics	Frequency	Percent
**Age**		
< =35 years	123	30.5
36- 49 years	179	44.4
> =50 years	101	25.1
Total	403	100
**Marital status**		
Married	280	69.5
Divorce	63	15.6
Widowed	60	14.9
Total	403	100
**Educational level**		
Unable to read and write	230	57.1
Primary	71	17.6
Secondary	52	12.9
Diploma	39	9.7
Degree and above	11	2.7
Total	403	100
**Occupational status**		
Self employed	15	3.7
House Wife	199	49.4
Merchant	47	11.7
Government employed	46	11.4
Farmer	96	23.8
Total	403	100
**Residence**		
Rural	151	37.5
Urban	252	62.5
Total	403	100

### Prevalence of sexually transmitted infection

Among 403 cervical cancer suspected women, 67 (16.6%) (95% CI, 12.9–20.6) were found to be positive for STIs. Of the study participants, 36 (8.9, 95% CI = 6.2–11.9) were HIV positive, 10 (2.5, 95% CI = 1–4.2) were HBV positive, 4 (1, 95% CI = 0.2–2.2) were HCV positive, and 29 (7.2, 95% CI = 4.7–9.7) of were syphilis positive.

Of the STIs positive women, 12/67 (17.9%) had serological evidence of multiple infections. Among those with multiple infections, 9/67 (13.4%) were HIV- syphilis positive, 1/67 (1.5%) were HBV-syphilis positive, 1 (1.5%) were HCV-HIV positive and 1 (1.5%) were HCV-syphilis positive.

### Risk factors for the acquisition of sexually transmitted infections

Firstly, bivariate logistic regression analysis was done for all study participant (*N* = 403) and variables with *P*-value ≤0.2 were further tested by multivariable logistic regression analysis. History of multiple sexual partners, history of an STI, not using condoms, alcohol addiction were an independent risk factors for STI. Patients who had multiple sexual partners were three times more likely to develop STI compared to those who had no multiple sexual partner (Adjusted OR = 3.02, 95% CI 1.57–5.79, *P* = 0.001). Similarly, the odds of developing STI were 3.4 times higher among patients who had a history of STI compared to the counterparts (Adjusted OR = 3.38; 95% CI: 1.76–6.47, *P* = 0.00). Besides patients, who had a habit of drinking alcohol were 2.2 times more likely to have STI compared to those who were not (Adjusted OR = 2.2, 95% CI 1.07–4.5, *P* = 0.031) (Table 2).

**Table 2:** Risk factors of STIs among cervical cancer suspected at University of Gondar Comprehensive Specialized Hospital

Variables	STIs status		COR (95% CI)	*P*-vale	AOR (95% CI)	*P*-value
	Neg (N)	Pos (N)				
**Multiple sexual partner**						
No	197	20	1			
Yes	139	47	3.33 (1.89–5.87)	0.00	3.02 (1.57–5.79)	0.001*
**Tattooing**						
No	126	18	1			
Yes	210	49	1.63 (0.91–2.93)	0.099	1.05 (0.54–2.03)	0.9
**History of Abortion**						
No	244	39	1			
Yes	92	28	1.9 (1.11–3.27)	0.020	1.5 (0.79–2.85)	0.217
**Tooth extraction**						
No	223	29	1			
Yes	113	38	2.59 (1.52–4.41)	0.00	1.4 (0.72–2.71)	0.33
**History of STI**						
No	264	32	1			
Yes	72	35	4 (2.32–6.92)	0.00	3.38 (1.76–6.47)	0.00*
**Using Condom**						
Yes	61	4	1			
No	275	63	3.5 (1.23–9.96)	0.019	4.99 (1.5–16.16)	0.007*
**Alcohol addiction**						
No	148	14	1			
Yes	188	53	2.98 (1.59–5.58)	0.001	2.2 (1.07–4.5)	0.031*
**Smoke Cigarette**						
No	307	54	1			
Yes	29	13	2.55 (1.25–5.21)	0.01	1.34 (0.51–3.56)	0.55

Key-*statically significant (*P* ≤ 0.05), 1 = reference value, *pos* positive, *Neg* negative, *N* number, *COR* crude odd ratio, *AOR* adjusted odd ratio, *CI* confidence interval

## Discussion

The burden of sexually transmitted infections is high among cervical cancer suspected women this is due to the fact that HPV is a sexually transmitted infection and they share a common route of transmission and risk factors. This study has sought the seroprevalence of STI, HIV, HBV, HCV, and syphilis infections and contributing factors for STI among cervical cancer suspected women attending at the University of Gondar Comprehensive Specialized Hospital.

The overall prevalence of STIs in the current study was 16.6% (95% CI = 12.9 to 20.6%). The finding of this study was higher than studies conducted in Gondar which was 10.5% [9] and 11.7% in India [10]. The high prevalence of STIs among cervical cancer suspected women is due to the fact that cervical cancer-causing virus and sexually transmitted infections share a common route of transmission and risk factors.

The present study showed that the prevalence of HIV was 8.9% (95% CI = 6.2 to 11.9%). It was higher compared to the study conducted in Cameroon which was 4.2% [11] and 4.1% in Nigeria [12]. The variation could be due to the fact that cervical cancer suspected women are more risk group for STIs namely HIV. An individual infected with one STIs become a high risk to be infected by HIV. Human Papilloma Virus causes inflammation around the genital area which increases the concentration of “activated” immune cells. Although the inflammatory response is meant to help fight the HPV, HIV likes to infect some of these recruited immune cells, also known as CD4 cells. Therefore, if someone has an STI (HPV) in the genitals and that area is exposed to HIV, the higher concentration of “activated” CD4 cells facilitates HIV infection, replication, and spread throughout the body [13]. In contrast to this; higher prevalence observed in South Africa, 14% [14]. This may be due to differences in socio-cultural behavior.

The seroprevalence of HBV was also noted in the current study and it was 2.5% (95% CI = 1.0 to 4.2%). Comparable results were also reported in Ethiopia ranged from 3 to 3.8% [15–17]. However, the higher prevalence was reported in Gondar (4.7%), Dessie (4.9%), and Congo (5.9%) [18–20]. This difference might be attributable to differences in risk for HBV acquisition and the impact of education level. In contrast to this, the lower prevalence was reported in India (0.9%) [21] and Iran (0.7%) [22]. The discrepancies might be a result of differences in sampling population, methodological difference, and geographical variation.

The prevalence of HCV in this study was also determined and it was 1% (95% CI = 0.2 to 2.2%). The finding of this study was in line with studies conducted in the Gondar Health Center 1.3% [9], 0.26% North-west Ethiopia [23]. However; a higher prevalence of HCV was documented among the attendants of voluntary counseling and testing for HIV in Ethiopia 9.1, 6.0, and 4.3% in Hawassa, Mekelle, and Adwa, respectively [24–26]. The variation is due to differences in risk behavior, sampling size, and study population. Besides, HCV infection is majorly acquired via blood transfusion other than other STIs.

Moreover, the prevalence of syphilis among cervical cancer suspected women in this study was 7.2% (95% CI, 4.7–9.7). The results from this study were in agreement with the report in South Africa 5% [14], 6.4% in North Tanzania [27], 5% in Nigeria [12]. Studies with lower prevalence compared to our findings include; 2.3% in Gondar Health Center [9] and 2.9% in Addis Ababa [28]. The high prevalence of syphilis among cervical cancer suspected women is due to the fact that cervical cancer-causing virus and *T. palladium* have a common route of transmission and risk factors. On the other hand; an extremely higher prevalence (17.4%) was reported in Cameroon [29] and 10% in South Western Nigeria [30]. The variations could be largely due to geo-cultural differences and the decrease time trend of syphilis is also claimed in Ethiopia.

Lastly, the present study was also aimed to identify independent risk factors of STIs. The distribution of STIs was significantly higher among alcohol user women than their counterparts in the current study. In brief, patients, who had habit of drinking alcohol, were 2.2 times more likely to have STI compared to those who were not. This might be due to the fact that alcohol use is one of the common factors that lead people to exhibit risky sexual behavior and lowers a person’s inhibitions, which may influence their sexual behavior, and thus a compromise safe sex practices (sex without condom) [31–33].

Additionally, the prevalence of STI was significantly higher among women who had a history of STIs (*P* < 0.001) which conforms to previous studies conducted in Jimma [15] and Peru [34]. Moreover, the prevalence of STI was also significant and three times higher among the study subjects who had a history of multiple sexual partners compared to their counterparts. This is due to women who have multiple sexual partners who had a chance of getting sex with an infected person.

Furthermore, STI prevalence was five times higher among study participants who have not used condoms compared to their counterparts. Without any doubt, the condom is one of the preventive strategies against STIs. Evidently, consistent and correct use of condoms is 90% effective in reducing HIV, up to 66% effective in reducing syphilis [35]. Similarly, studies have done in New York and Southwestern China support the advantage of condoms in preventing STI [36, 37]**.** In this study, however, STI did not show any correlation with the history of blood transfusion, tattooing, surgical operation, smoking, and genital mutilation in the present study. This is similar to previous studies in Ethiopia [16, 38].

## Conclusion

This study shows the prevalence of sexually transmitted infections was generally higher among cervical cancer suspected women. Having multiple sexual partners, not using condoms, alcohol addiction, and history of STIs found to be independent risk factors for sexually transmitted infections.

Since STIs are major health problems in cervical cancer suspected women, all cervical cancer suspected women should be screened for STIs (syphilis, HBV, HCV), increase awareness of people on modes of transmission of sexually transmitted infections and educate people how to prevent sexually transmitted infections could help in reducing the burden of STIs.

## Data Availability

All data generated or analyzed during this study were included in this article.
